# Strategies implemented by South African teachers to ensure continuing mathematics education during COVID-19

**DOI:** 10.1007/s11858-022-01408-9

**Published:** 2022-09-12

**Authors:** Pamela Vale, Mellony Graven

**Affiliations:** Rhodes University, Makhanda, South Africa

**Keywords:** Covid-19, Home learning, Pandemic teaching strategies, Primary teachers

## Abstract

The COVID-19 pandemic continues to present severe challenges to the education sector more than 2 years after the first case was detected. We explore the strategies South African teachers used to support continued mathematics learning at home during the COVID-19 pandemic across different stages of the response to it and across different contexts. We explore these strategies, first, in relation to the initial shift to emergency remote teaching when learners needed to be reached in their homes under severe lockdown regulations, then through the gradual reopening of schools later as regulations were relaxed. Activity theory informs our perspective on the way in which teachers worked to mediate learning across school and home activity systems. Twenty-five Grade 4–7 mathematics teachers from the Eastern Cape responded to two questionnaires given at the start of the COVID-19 lockdown period and during the phased return to school. Questions focused on the strategies used to support mathematics learning. The results show that strategies focused on engaging caregivers through various technologies and getting resources into the home. WhatsApp, a free internet-based messaging service, was the most frequently used communication app across all types of schools for both messaging parents and sending instructional material and support in the form of videos, pictures and text messages. Department issued workbooks and teacher designed work packs were key resources sent to homes. Differences are evident in the range of use of different technologies across wealthier and poorer schools. Primary teachers’ voices inform possible ways forward for the purpose of managing young student mathematical learning gaps and meeting ongoing learning needs.

## Introduction

South Africa is characterised by extreme economic and educational inequalities stubbornly persisting along racial lines. The 2003 TIMSS study showed that South Africa had the largest variations in schools between rich and poor. The average scores of learners in historically African schools were close to half of those in historically White schools (Reddy, 2006). This inequality continues to date as reflected in the most recent TIMSS 2019 results (Reddy, 2021). During apartheid, South African society was classified into four racial groups, namely, White, Coloured (of mixed race), Asian and African (of Asian and African origin respectively). These racial categories extended into the education system which had four segregated Departments of Education with differential funding (the White department received the most while the African received the least). The bi-modal performance distribution in education is particularly clear in mathematics, where previously White schools tend to perform well in international comparisons while historically African schools compare dismally (Reddy et al., 2015). Soudien et al. (2022) argue that the learning losses due to COVID-19 will be higher for mathematics than reading, and that “the achievement gains made since 1994 would probably revert to the achievement levels recorded in TIMSS 2015, a loss of 5 years of learning [and] [a]dditionally, the effect of the pandemic on the education system will widen existing inequalities” (p. 317).

COVID-19 continues to present severe challenges to the education sector in South Africa more than 2 years after the first case was detected. Hoadley (2020) suggested that children lost up to 42% of the official school days in 2020. In addition, rotational systems (for social distancing) adopted once schools reopened (continuing for many schools into 2022) halved learner attendance on any one day.

South Africa instituted five different alert levels, with varying restrictions. At Level 5 (from 27 March 2020), all were confined to their homes and schools were closed. Under Level 4 (from 1 May 2020), schools were gradually reopened for staff to return. From Level 3 (from 1 June 2020) and throughout Level 2 (from 18 August 2020), learners were gradually phased back into schools, grade by grade, with strict social distancing rules which were prohibitive of a full return. Prolonged educational disruptions require novel strategies to link home and school and innovative teaching strategies. The loss of teaching and learning time continued into 2022 and the effect is likely larger than a tally of days lost would reflect (van der Berg et al., 2020). Furthermore, learning losses will “map onto existing social and educational inequalities” (Hoadley, 2020, p. 8) due to vast disparities in access to educational resources in the home.

To inform possible ways to address these gaps within a continuing pandemic context we investigated the following question: What strategies have primary teachers used to ensure continuing mathematics learning during the COVID-19 pandemic? We focus particularly on how Grades 4–7 teachers used different mediums of communication and mediating artefacts in their intention to support continued learning in student homes. The insights gained from teachers indicate opportunities for building on these strategies moving forward.

## Literature review

The abrupt closure of schools due to COVID-19 necessitated a rapid re-imagination of how to deliver quality mathematics education resources to children isolated at home. Teachers had to innovate at speed to adapt to this new home learning context. Hodges et al. (2020) proposed the term “emergency remote teaching” to define those initial responses and strategies used in the continuation of teaching and learning. The objective of this emergency remote teaching is “not to re-create a robust educational ecosystem but rather to provide temporary access to instruction and instructional supports in a manner that is quick to set up and is reliably available during an emergency or crisis” (p. 13). This instruction is reliant on access to the resources necessary to set up instruction and instructional support. However, the “teacher-resources relationship is complex and not always optimal, particularly in disadvantaged educational environments where resources are constrained” (Adler, 2021, p. 1207), as is the case for most schools in this study.

### Use of digital resources

The pandemic necessitated using digital resources not previously used for teaching or communicating with families. Teachers had to further manage the challenge of inequitable access of learners to resources. Barbour et al. (2020) emphasised that this sudden use of digital resources contrasts with learning experiences designed from their inception to be online. There is a qualitative difference between a learning experience designed to be online, and a more ad hoc enactment that necessarily occurred at “unprecedented and staggering” speed (Hodges et al., 2020, p. 1).

Graven et al. (2021) argued that COVID-19 sped up the digital transition in education globally, but cautioned that increased access to quality mathematics education resources brings “enormous unevenness in systemic and individual preparedness to optimise these opportunities. This unevenness plays out across countries and within countries and there is concern that the digital divide could exacerbate inequities across gender/social-class/race” (p. 1). Clark-Wilson et al. (2020) noted a range of factors and obstacles influencing whether teachers (in pre-COVID-19 times) used digital technology for teaching. These include “their beliefs about and attitudes towards the technology, and attitudes towards the nature of mathematical knowledge and how it should be learned” (p. 1223). We would add that teacher and learner access to, and familiarity with, technology is also a key factor. Changing teaching to incorporate digital technologies is challenging (Drijvers et al., 2013) and teachers vary in their familiarity with digital resources. While some may embrace assimilating these into their teaching, others may not have the skills and confidence to do so (Drijvers et al., 2013). This said, the urgency with which communication and teaching and learning demanded online migration due to COVID-19, resulted in many teachers (irrespective of skill and confidence levels) adopting these technologies for the first time.

Remillard et al.’s (2021, p. 1331) study across four countries showed that “participation in social media and resource sharing altered the nature of and ways teachers participated in their own professional learning” and argued that digital resources have transformative potential in teaching and learning. They described three major domains of digital resource use, namely, class instruction, learner practice, and professional learning. We add to these their use in communication with families. During COVID-19, communicating with caregivers of young learners became critical to enabling continued mathematics learning during periods of lockdown. Trouche et al. (2020) usefully distinguished between mathematical software, programming applications, and “organising, sharing and communicating applications” (p. 1245). It is the latter technology that dominates in the strategies that primary teachers in our study used. These allowed teachers to communicate with parents and provide learning support.

### Studies of teacher responses to the pandemic teaching context

Several studies have emerged on teacher experiences of the pandemic and the measures they implemented to continue mathematics teaching and learning, particularly in secondary school and university contexts. For example, Drijvers et al.’s (2021) investigation of 1719 secondary mathematics teachers in Flanders, Germany and The Netherlands found a significant increase in the use of video conferencing and teacher confidence in using technology. Similarly, Barlovits et al.’s (2021) study of 248 mostly secondary German and Spanish teachers found that video conferencing was the preferred means of task discussion and was frequently used for teaching while e-mail and online learning platforms were often used for task assignments. On the other hand, social media and messenger services were used only by a small percentage of teachers. This contrasts with our findings in which primary teachers indicated messenger services as the prevalent means of connecting with students during the pandemic. Free online messenger services were however found to be used by a majority of Slovakian secondary mathematics teachers, along with e-mail and use of a web page in Csachová and Jurečková’s (2021) small scale study. These teachers mentioned problems with slow internet connectivity and conditions unsuitable for online education as challenges working against use of other technologies. The challenge of internet coverage as well as inequitable access to technological equipment for mediating distance learning was similarly noted in a ninth-grade class of rural Brazilian learners (Carius, 2021).

Similarly to Brazil, most South African learners are from poor socio-economic backgrounds with limited access to the internet and technologies for accessing online learning opportunities. In Chirinda et al.’s (2021) questionnaire and telephonic interview-based study of the strategies employed by 23 Grade 12 South African mathematics teachers during lockdown teachers reported the need “to be innovative” (p. 7) in reaching learners in their homes. The online platforms used by these teachers included Zoom, YouTube, Facebook Live and WhatsApp. Findings in our study indicate WhatsApp as the dominant platform used because teachers stated that learners did not have sufficient data to use other applications.

The above-mentioned studies all focused on secondary school learners using online questionnaires (though Chirinda et al. included telephonic interviews). We expect the dominance of questionnaires, as in the case of our study, was necessitated by the physical distancing required during lockdown periods, and continued distancing was recommended wherever possible in the post lockdown periods. While the above research provides findings on teacher experiences across contexts, we noted limited research involving primary school teachers. This is a gap in the research as the challenges of communicating with young learners at a distance are somewhat different (e.g., communicating with young learners at home necessitates greater parental involvement). Our study contributes to this gap in primary teacher experiences of managing pandemic challenges for supporting continued mathematics learning. Further, our research points to differences and similarities in strategies teachers in differentially resourced schools implemented, within a relatively narrow geographical area, thus illuminating the way in which inequity in pandemic responses plays out in relation to broader inequality within socio-political-economic contexts.

### Homework and parental involvement in mathematics education

Mathematics homework has not been a regular practice of learners in low SES schools in the Eastern Cape (Graven, 2018). Darragh and Franke (2021, p. 1) found that “mathematics homework is often unsuccessful or stressful for both parents and children and…tension exists between home and school in the learning of mathematics” and the quality of interaction between parents and learners in mathematics is influenced by parental mathematical ability. They note that changes in mathematics curricula and pedagogies mean parents are less able to draw on their own experience of learning when assisting their children. The pre-pandemic findings of Wadham et al. (2020) similarly revealed tension between parent and teacher perspectives on mathematics teaching and learning. In the South African context, where multiple curriculum changes have occurred in the past two decades, this is a relevant point. We note, however, Graven’s (2018) positive experience in implementing a ‘homework drive’ in Eastern Cape schools through provision of take-home workbooks. Teachers shifted from initially negative to positive comments about parental involvement and the mathematical value of homework at the end of the intervention. Similar shifts in perceptions of parents’ willingness and abilities to support their children’s learning in the home emerged in our data. For several teachers this experience pointed to opportunities for changing ways of organising “education when it does not need to be online anymore” as was suggested as an important theme for future mathematics education research (Bakker et al., 2021, p. 1).

## Methodology

This research was a qualitative, interpretive case study. We adopted a sociocultural perspective viewing reality as conditional on experiences and interpretations, and knowledge as socially constructed (Vygotsky, 2012). Aligned to this we sought the experiences of the teacher participants and foregrounded their voices in our analysis. Twenty-five Grade 4–7 teachers from 8 public schools in the Eastern Cape participated by answering two questionnaires. The learners in these grades ranged in age from 10 to 13 years. The questionnaires generated narrative responses of their experiences of, and the strategies used for, mathematics teaching and learning during the pandemic. Ethical clearance was received from the Rhodes University Education Faculty Research Ethics Committee. Participation was voluntary and participants could view questionnaires prior to agreeing to participate. Anonymity of the schools and participants was assured.

### Research context

The Eastern Cape, where this research is situated, is one of the poorest provinces in South Africa and has among the lowest educational performance of the nine provinces. Despite these challenges, mathematics teachers have shown ingenuity and resilience in their efforts to enable continued learning in homes as a primary site of learning during COVID-19. The participating teachers were all members of the Mathematics Inquiry Community of Leader Educators (MICLE) of the South African Numeracy Chair Project, Rhodes University. The project consists of university-based researchers partnering with local communities and schools in exploring sustainable ways forward for improving mathematics teaching and learning locally and beyond. The second author is the incumbent Chair while the first author was the project manager and the MICLE co-ordinator. The MICLE included 30 Grade 4–7 teachers across 8 schools meeting face-to-face bi-monthly from January 2019 until the pandemic lockdown in March 2020.

Systemic national education trends highlight links between poor mathematics performance and poor schools (where most learners are Black and English is not their home language) (Reddy, 2021). The inevitable deficit discourse of poor schools having persistent poor performance often leads to teacher blaming that can strip teachers, learners and families of their agency to innovate ways out of these challenges (Graven, 2014). Such studies must be balanced with studies of grounded experiences of teachers grappling to challenge these correlations within their historically (and continually) under-served schools and communities. In this paper we note the historical racial categorisation of our partner schools because differential educational and socio-economic resources of the school communities have enormous influence on what strategies are feasible. Across the different categories our partner schools have vastly different access to physical resources (such as physical classroom space, books, computers, teaching resources, printing/copying facilities, toilets, running water), human resources (such as low teacher pupil ratios and administrative support), and knowledge resources (such as teacher education levels and mathematics specific knowledge for teaching, and linguistic and social capital resources). Each of these resources impacts what is possible for enabling continued learning in a pandemic context.

We worked with teachers in three of the four categories of schools, that is, formerly White schools, so-called historically Coloured schools, and so-called historically African (rural and township) schools. In our area there were no historically Asian schools. We report the data in these historical categories because of emergent similarities among teachers in each category. Teacher responses aligned closely with the historically influenced resource conditions of schools. That said, the historical categories of the schools are not an indication of the racial composition of the schools—in all the schools in this study (and reflecting the provincial population) most learners are Black African learners speaking isiXhosa at home.

### Research approach

The theoretical framework guiding the research approach and analysis is Activity Theory taking as its point of departure Vygotsky’s (2012) triangular model that there is a transcending “complex mediated act” (p. 40) between a stimulus and a response. The triangular model of subject, object and mediating artefact represents an activity system. In our study our subject, “the individual whose agency is chosen as the point of view in the analysis” (Engeström, 1993, p. 67), is the teacher. The object is mathematics learning and the mediating artefacts are the tools that teachers use through “draw[ing] on existing tools and us[ing] cultural-historical resources to create new tools with which to engage, enact and pursue the object of their activity” (Foot, 2014, pp. 335–336).

**Fig. 1:**
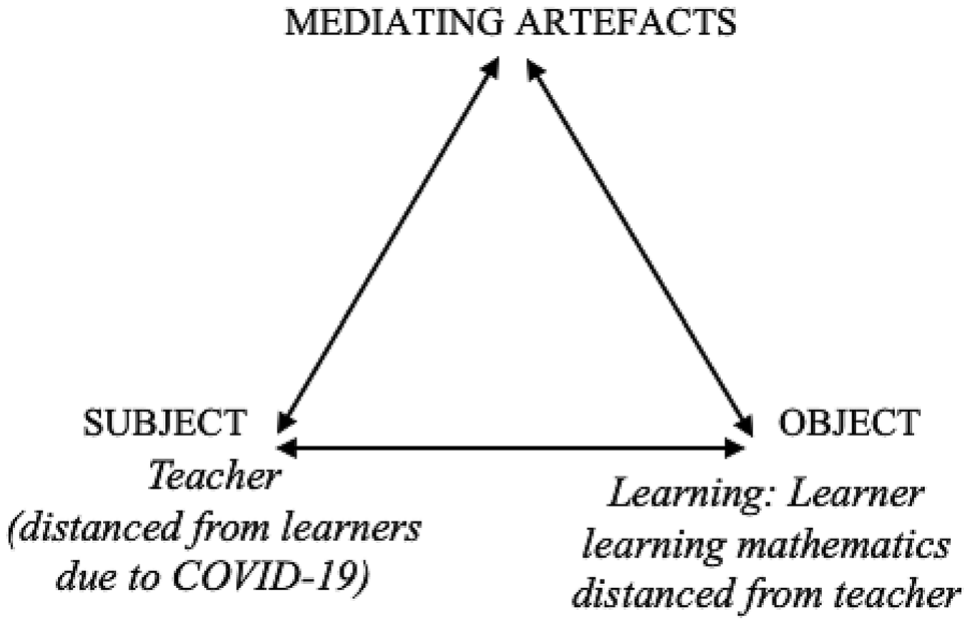
The school activity system at the beginning of COVID-19 lockdown

Engeström (1999) explained that activity systems are discontinuous and are neither stable nor harmonious—there are “failures, disruptions and unexpected innovations” (p. 32). There is incremental change, as well as “crises, upheavals and qualitative transformations [and] it is also a creative, novelty producing formation” (p. 70). The activity systems of schools were severely disrupted from 27 March 2020. The sudden closure of schools meant that the teachers and learners were isolated from schools. As a result, teachers had to manage facilitating learning in a new activity system—learners’ homes. They had to navigate connecting with these home spaces during lockdown and in the phased return to school, as well as in the full return as many learners were still kept home by parents. The focus in this study is the teacher as the subject and the strategies used in enabling the object of student mathematical learning. Figure 1 represents the school activity system at the start of the lockdown period.

The mathematics teacher is the voice (subject) that is represented in this study. We continued engaging with the MICLE teachers during the pandemic, on the basis that building joint interest between ourselves, teachers and parents in enabling continued mathematical engagement of learners at home and during the pandemic was both possible and important. While parent/caregiver and learner voices are important to understanding the full range of learning enablers and challenges in the home, we restricted our research to the teachers’ strategies for enabling continued mathematics learning in the home, largely due to the constraints in gathering data from parents and learners during pandemic conditions. Even in the period when the lockdown was lifted and there was a phased return of learners to school (described below) there were limitations on who could enter schools. Through our teacher community we had well established relationships with teachers and an already functioning WhatsApp group that we used for communicating with teachers. This enabled us to invite their participation in our research. Through the teacher data we gained insight into the strategies (means of mediating learning) and mediating artefacts used in homes and their experiences of challenges and opportunities in relation to these.

The question we addressed was as follows: What strategies have teachers used to ensure continuing mathematics learning during the COVID-19 pandemic? We focused particularly on how teachers used different mediums of communication and mediating artefacts in their intention to support continued learning in student homes.

### Data collection

Data collection through questionnaires occurred at two points, November 2020 and August 2021. As few MICLE teachers had e-mail and access to online platforms, we chose pencil and paper written questionnaires that we delivered to and collected from school offices. The questions we asked focused on eliciting the strategies the teachers used to ensure learners’ continued mathematics learning within pandemic restrictions.

While we had hoped to conduct interviews with teachers once pandemic restrictions were lifted, the ongoing need for continued social distancing throughout 2020 and 2021 made face-to-face interviews not possible. We considered telephonic interviews, however, our awareness of the stress teachers were under (ad hoc personal communications with teachers and from our reading of their responses to questionnaires) led us to use only questionnaires. Questionnaires are a convenient and economical means of reaching a geographically dispersed sample (Creswell, 2011) and allowed teachers to answer them in their own time. We note most research we reviewed on mathematics teacher experiences during COVID-19 similarly used questionnaires presumably because of their suitability in the pandemic context. Table 1 gives the questionnaire items. All items had an open response format.

**Table 1:** Questionnaire items

Questionnaire 1 (November 2020)
What are your greatest concerns in terms of learners’ mathematics learning this year?
What strategies, if any, did your school implement during the school shutdown periods?
What strategies, if any, did you implement for your learners’ continued mathematics learning during Stage 5 lockdown?
What strategies, if any, did you implement for your learners’ continued mathematics learning during Stage 4 lockdown?
What strategies, if any, did you implement for your learners’ continued mathematics learning during Stage 3 lockdown?
What strategies, if any, did you implement for your learners’ continued mathematics learning during Stage 2 lockdown?
What strategies, if any, did you implement for your learners’ continued mathematics learning during Stage 1 lockdown?
Did you use technology to support you in managing the challenges of continuing mathematics education during the pandemic? How did you use this technology?
Do you have any suggestions or recommendations for support for managing some of the lost mathematics learning time?
Are there any other experiences of the current pandemic that you would like to share?
Questionnaire 2 (August 2021)
At the start of learner rotation (in 2020) what strategies, if any, did you use to manage teaching and learning in the classroom and at home?
Are there new strategies that you are using in 2021 that you did not use last year?
Are there strategies that you have developed as a result of COVID disruptions that you might continue using even after schooling returns to ‘normal’?
COVID could disrupt schooling for a number of years. If you imagine an ideal world in which teachers are offered any and all resources they would like in order to support teaching and learning during such disruption periods, what resources would these be?
Explain why these resources would be helpful and how you would use them
Please share anything else about your experiences of teaching mathematics so far in 2021

The types of schools have been coded as HA (historically African), HC (historically Coloured) and HW (historically White). The HA and HC schools in the sample are non-fee-paying schools situated in low SES communities (with HA schools in lower SES communities than the HC schools). The HW schools are all fee-paying schools situated in wealthier areas. Participating teachers included 7 from 2 HA schools, 10 from 3 HC schools and 8 from 3 HW schools. We thus had 25 teachers in total.

### Data analysis

Our first and second questionnaires connect with Barbour et al.’s (2020) two phases of educational responses to the pandemic. Questionnaire 1 came at the end of Phase 1 that refers to the “rapid transition to remote teaching and learning” (Barbour et al., 2020, p. 3), in other words Emergency Remote Teaching (Hodges et al., 2020). Our second questionnaire links with Phase 2, which involves institutions adjusting the initial response to allow for more equitable access to learning resources, and the ‘(re)adding’ of basics (Barbour et al., 2020). In South Africa ‘(re)adding basics’ became possible upon the reopening of schools and the gradual return of learners to school, albeit on alternate days. As these two phases link to our two data collection periods we used these to structure our data. Thus Phase 1 data speak to responses about emergency remote teaching strategies in the initial 10-week school closure from 27 March to 31 May 2020 (encompassing Levels 5 and 4 of lockdown). Phase 2 data speak to responses about strategies used during the gradual re-opening of schools from June 2020 to August 2021 (i.e., from Level 3 of lockdown).

During our first reading of our data four broad strategy themes emerged: engaging parents, using technologies, distributing work, and scheduling extra classes. These formed the codes used in our first analysis of the data. Each response was coded separately by the authors according to one of these themes. While there was agreement in our coding of the data, this initial coding process highlighted the need for greater differentiation in the codes for technology and work distribution. This led to six strategy themes and a recoding by the first author according to these codes: using WhatsApp, using other technologies, distributing work packs, providing homework,^1^ and scheduling extra classes. The second author confirmed this coding. To ensure anonymity, in the coding of each response schools and teachers were assigned numbers, for example, HA2.1 is teacher 1 from historically African school (HA) number 2.

## Results

We begin with overview data of teacher responses of their and their schools’ strategies for continuing mathematics education through various levels of lockdown. Thereafter we reflect on the emergent themes and consider these against the three school categories.

Initial processing involved documenting when the schools and teachers began employing strategies for continuing mathematics learning, which indicated variation across schools. Similar variations in when schools returned to full attendance were noted. For all schools, learners initially attended school on a rotational attendance model, by having only 50% of the class present each day, and some schools were still working on this rotational system at the time of final data collection (31 August 2021). Schools differed in when they returned to full daily attendance as well as when they began implementing strategies for continued learning as shown in Table 2.

**Table 2:** Start dates for implementation of strategies and end dates of rotation

School	Strategies employed from	Rotation ended or continuing
Historically African (7 teachers)		
HA1	Level 4 (1 May 2020)	March 2021
HA2	Level 3 (1 June 2020)	Continuing^a^
Historically coloured (10 teachers)		
HC1	Level 4 (1 May 2020)	Continuing^a^
HC2	Level 3 (1 June 2020)	Continuing^a^
HC3	Level 3 (1 June 2020)	Continuing^a^
Historically White (8 teachers)		
HW1	Level 5 (27 March 2020)	15 Feb 2021
HW2	Level 5 (27 March 2020)	26 July 2021
HW3	Level 5 (27 March 2020)	26 July 2021

^a^Rotation was continuing at the end of data collection (31 August 2021).

Level 5—hard lockdown; Level 4—only teachers returned to school; Level 3—phased rotational return of learners to school

### Broad emergent themes

Engaging in dialogue with parents, mentioned by 80% of the teachers, is key to enabling learning in homes. It is the primary strategy all teachers used to support the object of learners’ learning mathematics (even while there was unevenness in reach across learners linked to different resource constraints in homes). Using WhatsApp and other technologies, distributing work packs and providing extra homework represent the innovative mediating artefacts that teachers used to support learning in homes. The extra classes were a measure implemented in schools when learners returned to school.

WhatsApp, an online messaging platform, was the most frequently mentioned technological tool used by all teachers for both information messaging and ‘mediation’ of content to be addressed at home. As noted above we thus gave it a separate code to ‘other technologies’. Some teachers, particularly in wealthier schools, also mentioned Zoom and Microsoft Teams (for synchronous video conferencing), YouTube (for asynchronous explanatory videos), Google Classroom (for facilitation of file sharing) and Facebook (for asynchronous sharing of information and resources) and more broadly, ‘the Internet’. After identifying these themes, a raw count of the number of times each was mentioned as part of teachers’ responses was done, disaggregated according to school type. Table 3 summarises these data.

**Table 3:** Number of mentions by number of teachers per identified theme

	Parents	Technology		Home activities		Extra classes
		WhatsApp	Other technology	Homework	Work packs	
HA (n = 7 teachers)	12 (4 teachers)	6 (3)	2 (2)	5 (5)	0	6 (6)
HC (n = 10)	30 (10)	29 (10)	3 (3)	35 (10)	16 (8)	3 (3)
HW (n = 8)	24 (6)	24 (8)	18 (6)	11 (6)	20 (7)	3 (3)
Total	66 (20)	59 (21)	23 (11)	51 (21)	36 (15)	12 (12)

Table 3 indicates interaction with, or involvement of, parents was mentioned the most (66 mentions from 20 of the 25 teachers). For example, “communication with parents has definitely helped” (HW2.2) and “the parents are involved” (HA1.3). The majority of the excerpts referencing parents were either neutral in tone (e.g., “work was sent to parents”, HW3.2) or indicated positive engagement with parents (e.g., “parental support played a big part in making sure…homework was done” (HW2.2), and “parents participate[d] very well…[they] are involved and explained to their learners the work” (HA1.3)). There were 6 examples of parents mentioned in a less positive light, most referencing parents’ reluctance to send their children back to school, e.g., “some parents decided to keep their children at home” (HC2.1).

Comments about WhatsApp appeared the second-most frequently (59 mentions from 21 teachers) and noted the way it conveniently supported communication with parents and learners [e.g., it was the “cheapest and the most convenient communication” (HC1.2)]. Low cost (and thus greater prevalence in homes) likely explains the higher frequency of WhatsApp in all three types of schools compared to other forms of technology. The use of ‘other technology’ is mentioned far fewer times and mostly by teachers from historically White (HW) schools.

Assigning extra homework was mentioned by 21 teachers with the third highest frequency (51 mentions) and distributing work packs for use at home had the fourth highest frequency (36 mentions from 15 teachers). ‘Extra classes’ represents the lowest frequency, though almost all teachers from historically African (HA) schools mention these extra classes.

### Teacher strategies used in historically African (HA) schools

In this section we reflect on teacher narratives in HA schools. Neither of the HA schools implemented co-ordinated measures of continuing education during the first (March 2020) lockdown Level 5, though one teacher (HA1.2) noted informal Whatsapp and phone calls with some learners: “There is nothing formal that I did because not every learner had access to the internet, but I did what I could with those that had access.”

HA1 began engaging with learners and their families under Level 4 (May 2020) when only teachers were permitted back to schools. The South African Department of Basic Education [DBE] issues workbooks for mathematics and these were sent to learners’ homes and became the key artefacts for home mathematics activity. Teachers stipulated the workbook pages learners needed to complete, communicated to parents via Whatsapp.

Teachers expressed awareness of inequities in the reach of their strategies. Information did not reach all homes, as “not all learners have cell phones for educational purposes” (HA1.1). Teachers at HA2 stated this inequity as the reason their school did not implement any coordinated strategies during Phase 1: “most parents don’t have cell phones so it was impossible to keep learners busy…we only start teaching when the learners return to school” (HA2.2). HA2 waited for their learners to physically return to school (Level 3, Jun 2020) before implementing measures to support continuing learning at home required by the rotational return system.

A major change occurred in both home and school systems in Phase 2 when the COVID-19 regulations were relaxed sufficiently to allow learners to return to school. This signalled the beginning of more co-ordinated interaction with homes for both schools. Learners were again a part of the school activity system. In addition to learners being present in the school community, the DBE appointed education assistants in both HA schools. These assistants were placed in schools to provide in-classroom support to relieve teachers of COVID-19-related and administrative duties. In Phase 2, the mediating artefacts oriented towards mathematics learning and catch-up of lost learning included the following: extra classes, extra homework, WhatsApp, and the DBE workbooks (now for home). In addition, Facebook messages were mentioned by one teacher.

HA1 maintained their strategy of referring learners to their DBE workbooks until the grade returned to school. During Phase 2 all teachers in the school began using WhatsApp as a means of communicating with parents and learners. Facebook was used by the school as a platform to broadcast more general messages to the school community. WhatsApp was used beyond messaging as a pedagogic device by 2 teachers. HA1.2 reported that “learners were able to contact me via WhatsApp, we were able to discuss and send pictures of activities and answers on the WhatsApp group that we opened” and HA1.3 wrote that she created a WhatsApp group “to discuss homework tasks and help them to achieve work and explaining using WhatsApp”. This app had been reimagined as a space for teaching and learning within the home, and reports of teachers suggest it facilitated good bi-directional communication.

The two HA schools had different experiences in connecting with homes. HA1.3 wrote “parents participate very well…parents are in charge calling asking about the problem how to do it…the parents are involved and explained to their learners the work and check their books”. This teacher noted that some parents did not have smartphones and thus could not interact. However, the teachers in this school suggested that generally their school interacted productively with the majority of their learners’ homes. They suggested most learners were doing some mathematics and at least some learning was assumed to be occurring at home. HA2 teachers noted less successful interaction with the home activity system: “Parents, learners and teachers were very afraid…it was difficult to get learners, teachers and parents motivated” (HA2.3). This teacher explains that “learners who are struggling with no support from home are struggling more”. The school in this case did not manage productive interaction with homes to ensure continued learning in Phase 1. On the physical return of learners, both schools implemented extra afternoon classes to try to catch up work missed. HA1 also mentioned issuing extra homework to learners for use on the days they were not attending school, thus maintaining the link with homes beyond Phase 1.

A tension is evident in the teachers’ descriptions of teaching during Phase 2 across both schools. Even once all grades had returned to schools, learners attended on alternate days so that each teacher would have only 50% of their class present each day. Teachers mentioned that “[l]earners did not get enough time in terms of teaching and learning in most of the topics…I did not cover all the work as expected” (HA1.1) and “a lot of content is left out as time force you to do more in half the time” (HA2.3). However, they also noted an opportunity in this: “teaching less learners each day gives me a chance to understand their challenges… I am able to see their work within the given period…they have an opportunity to say their views or thoughts on a given topic” (HA2.4). HA1.1 adds that “since the number of learners is manageable…at least there is contact with learners so it is much easier than last year. There is one-on-one and they can ask questions and express themselves.”

### Teacher strategies used in historically Coloured (HC) schools

All three HC schools (with 10 teachers in our study) chose not to implement strategies to continue learning during lockdown level 5. One teacher explained this, writing that “most of our learners come from poor backgrounds and don’t have access to phones, internet etc.” (HC1.3). Another explained, “We did not implement any strategies due to the circumstances of households and where learners are living. Most learners suffered a lot during this pandemic” (HC2.3). However once teachers were permitted to return to school premises, HC1 began reaching out to learners in their homes through creating and distributing work packs for home use. “The educators were asked to prepare work for the learners to do at home. The parents had to pick it up from school on certain days” (HC1.1). The work packs were the key mediating artefact created by teachers on their return to schools and collected for use in homes. The expectation was that the learners would do the activities in the work pack, and parents would monitor and support this.

As schools gradually reopened for learners, so the mediating artefacts originating from schools increased. Teachers now reached out to learners in their homes. The mediating artefacts sent to the homes, as deduced from the teacher narratives, were the DBE workbooks and homework packs compiled and distributed by the schools. WhatsApp and Facebook were the two communication platforms used by the teachers to message parents. As “WhatsApp was the cheapest and the most convenient communication” (HC1.2), it was the platform used. “Via WhatsApp parents were given the work to be done” (HC2.4). A teacher from HC3 writes: “I even communicated with parents through WhatsApp…I will continue using WhatsApp and involving parents…parents are very helpful and concerned about the education of their kids” (HC3.1). HC3.1’s experience of working with parents in facilitating productive interaction between home and school was not shared by all teachers. HC1.3 wrote, “His mother collected the work, but she never helped or assisted him to complete the tasks…some parents did not assist their children with the homework activities” (HC1.3). Teachers in this study have, however, been more positive than negative about the role of the parents in supporting learners’ continuing mathematics education.

The strategy used once learners were back in the classroom for 50% of the days was to give “activities in the classroom with enough homework to keep the learners busy while at home” (HC1.2), especially for days they did not attend school. This highlights the need for effective interaction with the homes as in class “there was not enough time for in depth explanations of certain topics, concepts and skills as well as individual attention…it was about superficial explanations, giving activities and homework for extra practice” (HC1.1). Extra homework and extra practice were seen as essential for catching up lost learning time and key to continuing learning in homes. Parents were also “encouraged to look for maths activities in the newspaper [and] tv programmes” (HC1.3).

It is interesting to note the echo of the double-edged sword noted by the HA schools’ teachers on the 50% rotational system in HC schools. One teacher explains that “it’s better to teach and work with less learners…the 50% of attendance of learners is good, because educators can work with individual **learners when needed to do so” (HC1.2).**

### Teacher strategies used in historically White (HW) schools

The HW schools implemented measures to continue education in homes from the beginning of COVID-19 school closures (Phase 1) and reported continuing these same measures in Phase 2 (thus HW school teachers made no distinction between Phase 1 and 2). Furthermore, the range of strategies was far wider than those used in the HA and HC schools. This is unsurprising as these are the most resourced schools and communities of our sample (even while there were wide differences in the SES of learners in these schools). The strategies generally used relied on access to technology.

Reliance on technology is clear throughout the data. Teachers from all three HW schools used WhatsApp to send information to parents. This included sending ideas for mathematics games to play in the home and sending links to YouTube videos and other internet sites with supporting explanations of mathematics concepts, even including videos of themselves explaining concepts. WhatsApp was used “as a mode of teaching and communication” (HW2.1). Teachers also “sent links out to relevant lessons to help parents understand” (HW1.1), and bidirectional communication was established, with HW2.1 noting that “parents contacted me privately with queries or questions”. Teachers in HW1 also mentioned teaching lessons via Zoom and Microsoft Teams and using Google Classroom. Once teachers were allowed access to schools, teachers from all three schools (similar to HC schools) created work packs which were collected from the school by parents.

The emphasis placed on technological mediating artefacts excluded some learners as noted by one teacher: “using technology to teach became extremely expensive for both parents and myself as an educator. Parents that lost their jobs during COVID couldn’t keep up with WhatsApp lessons on a daily basis” (HW2.2). The “immense cost…on our personal budget” (HW2.1) is worth noting as teachers were not provided financial support for their data costs in using these strategies. Work packs would have allowed learners without access to technology the opportunity to continue mathematics work, but without the benefit of the explanations of the work that were available to learners whose home environment included internet access. “Helpful videos that reinforce a specific topic were shared onto the [WhatsApp] group to help learners who needed more consolidation” (HW3.1), but not all learners had access to this.

In Phase 2, teachers continued using work packs, and also assigned extra homework for the days learners were not at school. There were mixed comments on the effectiveness of this strategy. One teacher mentioned “parental support played a big part in making sure booklet homework was done” (HW2.2), but others noted that “children do not do their homework” (HW2.1) and “some learners have shown no interest” (HW1.1). HW2.2 commented about learner homes mentioning that some children live with grandparents who are unable to help with homework, and that “the way maths/concepts are taught is…different compared to parents’ education”, thus limiting the ability of the home to achieve the object of learners meaningfully engaging in mathematics. HW2.1 explained that learners were “unable to teach themselves [and] most times it was incorrect, thus [it was] challenging as a teacher to ‘unteach’ that incorrect method”. This issue echoes challenges noted by Darragh and Franke (2021) discussed earlier.

As with the HA and HC schools, teachers’ challenges and opportunities were noted concerning the 50% rotational attendance strategy. For example, while one teacher emphasised that “Maths NEEDS to be taught *every day*, not every second day” (HW2.1), another noted that “the two groups worked well” (HW3.1).

### Similarities and differences in strategies across school categories

Interesting similarities and differences are evident from the teachers’ narratives across historically African, historically Coloured and historically White schools. Tables 4 and 5 provide a view across contexts of strategies implemented to enable mathematics teaching and learning. The tables show similarities and differences in how teachers in these three differentially resourced schools and communities responded to pandemic challenges.

**Table 4:** Medium of communication used by teachers

	Historically African	Historically coloured	Historically white
Phase 1	Phone calls		
	WhatsApp	WhatsApp	WhatsApp
			Zoom
			Microsoft Teams
			Google Classroom
Phase 2	Phone calls		
	WhatsApp	WhatsApp	WhatsApp
	Facebook	Facebook	
			Zoom Microsoft Teams Google Classroom

**Table 5:** Mediating artefacts used to support home learning

	Historically African	Historically coloured	Historically white
Phase 1	DBE workbooks	DBE workbooks	
	WhatsApp messages	WhatsApp messages	WhatsApp messages
		Work packs	Work packs
			Extra homework Math games YouTube and other internet resources Google Classroom Zoom and Microsoft Teams lessons
Phase 2	DBE workbooks	DBE workbooks	
	WhatsApp messages	WhatsApp messages	WhatsApp messages
	Facebook posts	Facebook posts	
	Extra homework	Extra homework	Extra homework
		Work packs	Work packs
		Textbooks	
			Math games YouTube and other internet resources Google Classroom Zoom and Microsoft Teams lessons

Table 4 summarises the different technologies used by the teachers to communicate with parents and learners in Phase 1 and Phase 2. WhatsApp is seen as an important technological tool for communicating with parents across all school types. It is also clear that the historically white schools made use of a much wider range of communication tools to reach learners in their homes.

Table 5 summarises the mediating artefacts used in homes, as deduced from teacher narratives.

The tables highlight the way in which the differentially resourced schools and the communities they serve link to differences in the range of strategies of both communicating with parents and learners, mediating mathematical learning and activity and provision of activity resources. The most resourced schools, the HW schools, were able to utilise a far wider range of measures from Phase 1 to reach learners in their homes, with many of these being reliant on the use of technology. However, the tables also highlight interesting commonalities across schools, particularly Whatsapp and getting paper-based activities into homes (either workbooks (developed by the DBE), or work packs (developed by teachers), or both).

## Discussion

Most of the HA and HC schools delayed implementing strategies for continued learning until lockdown levels 4 and 3 (when teachers and learners respectively were allowed to return to school). These teachers noted the inequities in the reach of their strategies because of the differing resources in the home activity systems. These two school types were similar in the range of mediating artefacts utilised in working towards the object of the learners’ mathematics learning. The HW schools all implemented strategies for continued learning from the start of the first lockdown. These schools and the teachers employed a wider range of measures, largely reliant on various technologies, to reach the learner in their homes. This is unsurprising as they represent the most resourced schools in the sample. These teachers, however, similarly raised tensions of inequity of access in homes to the technologies used. While their schools are well resourced and situated in wealthier communities, most of their learners come to school from other much poorer areas. Notably teachers in HA schools mentioned WhatsApp and other technology (6 + 2 respectively) far less than those in HC (29 + 3) and HW (24 + 18) schools (see Table 3). The data indicate that even while WhatsApp is ‘cheap’ (the app is free, but data are needed) it is likely still out of reach for many parents from HA poorer schools. The ‘other technology’ mentioned by teachers in HA and HC schools were Facebook and more broadly, ‘the Internet’, while teachers from HW schools in addition included explicit mention of using YouTube, Microsoft Teams, Zoom and Google Classroom for synchronous and asynchronous mediation of learning.

Homework was mentioned more often in HC schools than in HA or HW schools, with 35 mentions against the 4 and 11 mentions for HA and HW schools respectively. HW schools made the most mention of technologies other than WhatsApp, with 18 examples. One teacher explained “It is hugely beneficial to have access to videos, games and other technological resources” (HW2.2). While this points to embracing digital learning opportunities it also highlights the way in which such opportunities link to resources available in schools and their communities.

Extra classes also emerged as a strategy for supporting recovery of lost learning, especially in historically African schools (mentioned by 6 of 7 teachers). One teacher explained that “to cover the time lost, we also had extra classes after school” (HA2.2). Another measure that emerged in two HC and all three HW schools was the creation and distribution of work packs. The HA schools used the DBE workbooks in a similar way. These paper-based work packs/books provided opportunities for written mathematical work at home. These had to be collected from school offices during the times learners were unable to attend school (Phase 1).

While this paper reports on a small-scale case study, key insights emerge from the primary teachers’ voices. Beyond providing insight into their responses to the COVID-19 emergency remote teaching response they suggest opportunities, grounded in their experiences of what is possible in their range of schools and linked communities, for moving forward with and beyond the pandemic.

Activity Theory allowed us to consider the school and homes as different though interacting systems of learning activity. We focused on teachers as the subject (whose point of view is in focus), learners’ ongoing mathematical learning as the object, and we researched the mediating artefacts that teachers said they used to provide learning opportunities in the homes during the pandemic. This was important as homes replaced classrooms as the primary sites for learning (at least in the initial stages of lockdown) and continued as key sites during prolonged rotational return to schools. While the teachers in our study, as with teachers in other studies, noted challenges in achieving mathematics partnerships and communication between the home and school, all emphasised the critical importance of communication with parents during the pandemic and they plan to continue to build on the communication systems established during pandemic restrictions into the future.

The transformative potential of technology noted by Remillard et al. (2021) was acknowledged across schools. For all teachers the minimum of internet connectivity with a basic device to access WhatsApp and other digital resources were noted as essential for enabling continued home-based learning. Of interest even in the poorest of schools and communities many families had access to WhatsApp which emerged in this research as the common denominator across all schools and the key means for enabling ongoing communication between teachers and caregivers. In a recent emergency meeting of the DBE (TDCM 9–10 November 2021 Special Workshop on the Learning Recovery Framework: Creating an enabling Environment for Teaching and Learning) WhatsApp was noted as a widely used means by which teachers are supporting learning in the continuing pandemic context that has many public schools still teaching on a rotational basis. This aligns with the findings of Chirinda et al. (2021) who noted WhatsApp as the most frequently used platform by South African teachers because it used less data than other applications. This result stands in contrast to the European findings (e.g., Barlovits et al., 2021; Drijvers et al., 2021) where videoconferencing technology was increasingly used while social media and messenger services, like Facebook and WhatsApp were infrequently used. Our research suggests the basic technology of WhatsApp meant teachers could communicate with parents to provide opportunities for ongoing mathematical activity in homes. We suggest that all caregivers, irrespective of their socioeconomic status, should have free access to a basic device and a data package that allows for continued school-home communication.

Of interest, a shift occurred from Phase 1 (hard lockdown) to Phase 2 (phased and rotational return of learners to school) in several teachers’ use of WhatsApp. In Phase 1 there was some reluctance among teachers in the HA and HC schools to use technology for communication due to equity concerns. Concerns about inequitable access to technology in learner homes is echoed in studies in wealthier and poorer contexts (e.g., Barlovits et al., 2021 in Germany and Spain; and Carius, 2021 in Brazil). The extension of the pandemic, however, resulted in greater willingness of teachers to engage in technological solutions with the intention of continuing to draw on these forms to communicate with homes (irrespective of the pandemic). This points to a powerful lever of change and opportunity emerging from the pandemic. In pre-pandemic times the need to engage parents and provide mathematics learning opportunities outside of the classroom has been argued though often low expectations about learners and their communities’ abilities to support learning in their homes resulted in provision of limited opportunities (Graven, 2014). Deficit discourses around low SES parents and learners are present across our research in low SES communities (Westaway & Graven, 2019) and yet when teachers have been pushed to challenge these assumptions (whether through interventions or pandemic related necessities) we see rapid transformation of assumptions (Graven, 2018). This change was captured by teacher HC2.1 who said in Phase 1 “we did not implement any strategies due to circumstances and where children stayed”, yet in Phase 2 she mentioned successful creation and use of a WhatsApp group for each grade. The continued pandemic challenges thus shifted teacher views towards the importance of providing opportunities for learning beyond the classroom. While some teachers initially gave reasons of inequity for not providing learners with these opportunities at home, by Phase 2 all teachers implemented strategies for such opportunities. Further research into the extent to which equity concerns may at times work against providing innovative learning opportunities for South African learners could be useful. When the hard lockdown was lifted, debates emerged in the press about whether wealthier independent and public schools that were able to provide for social distancing measures should be ‘allowed’ to re-open to learners when most schools could not. We do not explore such a debate here but note, from our experiences in our Makhanda community, the desire of parents and learners (across all categories of schools) for increased opportunities for mathematical learning in the home. The extension of the pandemic has provided a stimulus for home learning resource provision and better communication with (and flow of information to) learner homes irrespective of differences in home resources that support use of learning resources and communication with teachers and schools.

## Concluding remarks

We foregrounded teacher voices on strategies used to enable the extension of mathematics teaching and learning in homes. The strategies employed ranged from technological solutions for communicating with the parents and learners in their homes, to solutions oriented towards providing physical work packs or workbooks for learners to complete at home. In addition, extra lessons were scheduled in some schools once learners returned to school. These strategies varied across school types according to the resources available at the schools and in the immediate community. HW schools and teachers used more technologically focused (and diverse) strategies from Phase 1, while HC and HA schools were more delayed in their implementation of technological solutions though once teachers returned to school HC and HA school teachers used WhatsApp to communicate with parents in homes and continued this use during the phased rotational return of learners to school.

We note from the responses from the teachers the need to focus research and policy priorities on how to affordably leverage the transformative potential of technology for ensuring meaningful engagement of learners in mathematics activities at school and at home. All teachers shared a common vision that mathematics education should incorporate the engagement of learners in mathematics both at school *and at home* and believed that the effective use of technology and ongoing communication with caregivers was key to this.

An opportunity many teachers highlighted related to the 50% rotational attendance model. Across all three school types there was mention of the loss of teaching time that this represented, but also a strong message that the opportunity to teach a smaller group of learners was positive: “It's better to teach and work with fewer learners, the 50% of attendance of learners is good, because the educator can work with individual learners when needed to do so” (HC1.2). The fact that this ‘benefit’ emerged across school types points to an opportunity to reimagine how teaching might be to allow for these smaller classes. The employment of education assistants across public schools during the return to school has provided opportunity for continued systemic support that allows teachers opportunities to work with learners in smaller groups. The increased provision of ‘independent’ learning activity materials, either physical or digital, for school and home use, could be a positive aspect of the pandemic that enables conceptualising these as a key part of supporting student learning trajectories and learning agency in homes post pandemic. Education assistants could assist with monitoring and administering of independent learning activities while teachers spend their time focused on working with learners in smaller groups. Education assistants could also be employed to support after school independent learning activities (especially for those learners who may not be able to complete these at home). Further and larger national research could usefully probe teacher views on key resources needed for supporting learning in homes in a continuing or post pandemic context and ways in which the current system of teaching assistants might be usefully incorporated into continuing opportunities that have emerged as a result of insights gained from the pandemic.

A limitation of this study is the absence of parent and learner voices. We chose to focus on teacher voices and thus have data only on teacher views of learner and parent experiences. An avenue for future research would be to explore parent and learner experiences of continuing mathematics teaching and learning in homes. Our research is also limited by the choice to focus on teacher strategies for supporting mathematics learning in homes (i.e., the mediating artefacts used and provided in homes in the pursuit of enabling continued mathematics learning), to the exclusion of other elements of the home activity system that influence *how* mediating artefacts are used.

This research brings primary mathematics teacher voices about their strategies for teaching mathematics through the pandemic into a field that has largely included secondary teacher voices. Furthermore, it illuminates the way in which continued socio-economic inequalities across schools and school communities, that persist along racial lines in post-apartheid South Africa, impact on opportunities for embracing technological innovations by mathematics teachers. We have noted calls for increasing research into teacher experiences of technologies they are using to address pandemic challenges. This paper makes a contribution towards that call, and through including the voices of primary mathematics teachers points towards possible avenues for navigating the way forward.

## Footnotes

1 Homework refers to extra work assigned for days on which learners were not attending school. This was a measure implemented when learners started to attend school on a rotational basis.
